# Revisiting Caroli Syndrome in a Tanzanian Patient

**DOI:** 10.7759/cureus.6661

**Published:** 2020-01-15

**Authors:** Casmir Wambura, Munish Sharma, Salim Surani

**Affiliations:** 1 Internal Medicine, The Aga Khan University, Dar es Salaam, TZA; 2 Internal Medicine, Corpus Christi Medical Center, Corpus Christi, USA; 3 Internal Medicine, Texas A&M Health Science Center, Bryan, USA

**Keywords:** caroli disease, caroli syndrome, portal hypertension, liver fibrosis, cholangitis

## Abstract

Caroli disease and Caroli syndrome are two rare congenital diseases of the intrahepatic bile ducts. Caroli syndrome is characterized by the saccular dilatation of intrahepatic bile ducts associated with congenital hepatic fibrosis. It is rarely diagnosed in childhood. We hereby describe a case of Caroli syndrome in a young Tanzanian female who had abdominal pain and distension since childhood. Her history suggested the presence of portal hypertension possibly from congenital hepatic fibrosis. The diagnosis was reached based on ultrasound, computed tomography (CT) scan of the abdomen, and magnetic resonance cholangiopancreatography (MRCP).

## Introduction

Caroli disease refers to biliary ductular ectasia without any apparent hepatic abnormalities while Caroli syndrome is the more common variant in which biliary ectasia is associated with congenital fibrosis of the liver [1-2]. Congenital hepatic fibrosis refers to unique congenital liver histology characterized by bland portal fibrosis, hyperproliferation of interlobular bile ducts within the portal areas with variable shapes and sizes of bile ducts, and preservation of normal lobular architecture [1-3]. Caroli syndrome is a developmental anomaly and there are many theories explaining its pathogenesis. The most acceptable theory is related to ductal plane malformation at different levels of the intrahepatic biliary tree [4]. This condition results from an embryonic derangement of the bile ducts. As major bile ducts are affected, the result is Caroli disease; whereas, abnormality affecting small ducts results in congenital hepatic fibrosis.

## Case presentation

A 28-year-old Tanzanian lady presented with complaints of intermittent right upper quadrant abdominal discomfort and a huge swelling in the left upper abdomen since childhood. She is the fourth born of six siblings who are healthy. Her mother had noted a left-sided firm abdominal mass since childhood. Past medical history was significant for vomiting blood at the age of two years. At the age of 16 years, she was diagnosed to have anemia and had received transfusion. She denied history of recurrent fevers.

On examination, she was found to be lean, moderately pale and non-icteric. The liver was not palpable. The spleen was palpable to the mid-pelvis, 20 cm from the left coastal margin mid clavicular line. Other physical signs were unremarkable. Laboratory investigations showed white blood cell count of 8.7 x 10^3^ per microliter of blood, hemoglobin 9 g/dl, mean corpuscular hemoglobin (MCH) 21.4 pg per cell and platelets count of 36,000 per microliter of blood. Liver function panel did not reveal any abnormality. Prothrombin time was 11 secs, international normalized ratio (INR) 1, and activated partial thromboplastin time (aPTT) 31 seconds. Peripheral blood smear revealed microcytic/hypochromic picture. Erythrocyte sedimentation rate (ESR) was 5 mm/hr and C-reactive protein (CRP) was 1 mg/L.

**Figure 1:**
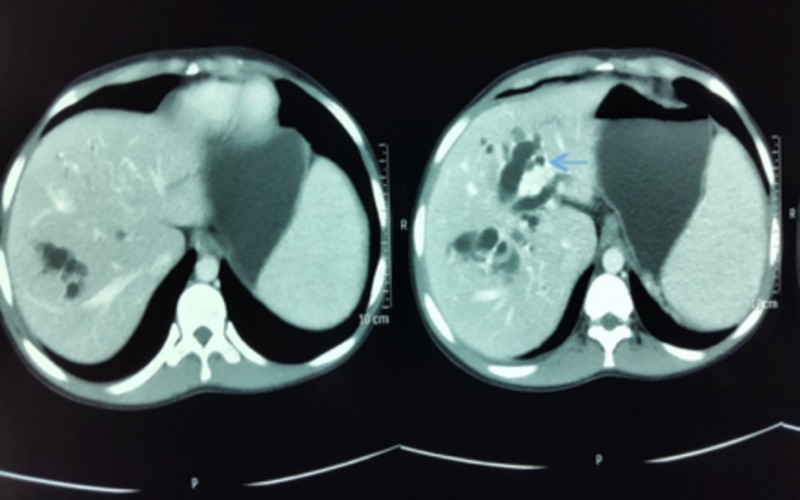
Intra hepatic biliary duct was grossly dilated and cystic (blue arrow) as seen in the computed tomography of the abdomen

**Figure 2:**
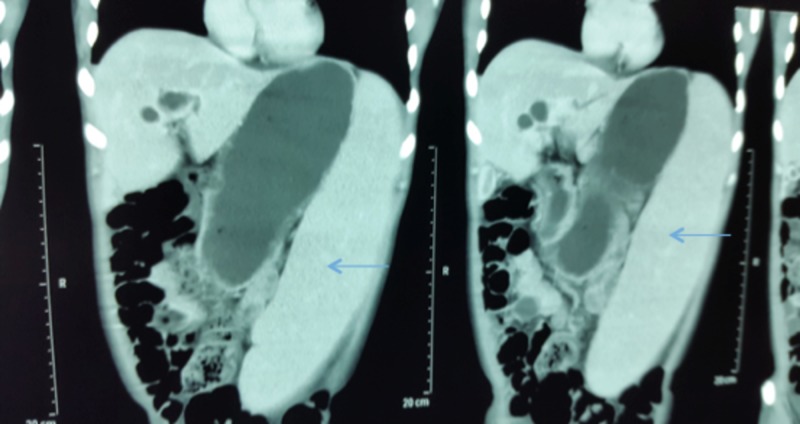
Computed tomography of the abdomen showing splenic enlargement (blue arrow)

Computed tomography (CT) scan of the abdomen showed a normal liver size with no parenchymal lesion. Intra hepatic biliary duct (IHBD) was grossly dilated and cystic (Figure 1). The common bile duct (CBD) was not dilated. The gallbladder was shrunken with increased wall thickness; the portal vein was dilated. There were multiple vascular collaterals at porta hepatis. The splenic vein was dilated to 15 mm. The spleen was grossly enlarged to 29 cm (Figure 2). There was a mild peritoneal free fluid. CT scan abdomen was consistent with non-obstructive IHBD dilatation and portal hypertension.

**Figure 3:**
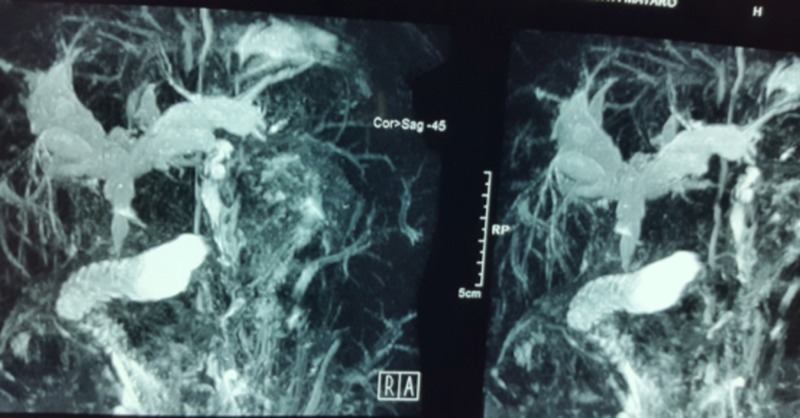
Magnetic resonance cholangiopancreatography (MRCP) showing dilated portal vein with multiple collaterals

Differential diagnosis included Caroli disease, primary sclerosing cholangitis, primary biliary cirrhosis, and partial extra-hepatic biliary stricture. Magnetic resonance cholangiopancreatography (MRCP) was performed, which revealed diffuse hypo density mild atrophic liver. Portal vein was dilated with multiple collaterals. Peripheral fibrosis, shrunken right lobe, and enlarged caudate lobe were revealed. The biliary system had massive IHBD dilatation, CBD dilated up to lower third with abrupt termination or narrowing over the short segment. Pancreatic duct was normal as seen in Figure 3. There was no intraluminal-filling defect. The gallbladder was poorly visualized. The spleen was massively enlarged up to 30 cm and there were dilated tortuous splenic collaterals (Figure 3). There were mild ascites. MRCP findings were consistent with severe IHBD dilatation, dilated CBD with distal stricture, liver cirrhosis, portal hypertension, and massive splenomegaly. Upper endoscopy was performed, which showed esophageal varices of grade II-III.

The patient underwent splenorenal surgical shunt and splenectomy and has been doing well at a follow up of more than a year till now.

## Discussion

Caroli disease results from an arrest in ductal plate remodeling at the level of the larger intrahepatic bile ducts. In contrast, Caroli syndrome develops when the full spectrum of bile duct differentiation is affected, such that smaller interlobular ducts are involved and congenital hepatic fibrosis is seen. Multifocal dilation may either be diffuse, affecting the whole intrahepatic biliary tree or may be confined to part of the liver [5]. It appears to be inherited in an autosomal recessive manner. Mutation in PKHD1, the gene linked to adult recessive polycystic kidney disease (ARPKD), has been identified in patients with Caroli syndrome [3-4,6]. 

Caroli syndrome presents a clinical syndrome, which is a combination of Caroli disease (bouts of cholangitis, hepatolithiasis, and gallbladder stones) and those of congenital hepatic fibrosis (portal hypertension) [3,5,7]. Clinical progression and presentation of Caroli syndrome is highly variable, and symptoms may appear early or late during life. Although present from birth, the disease usually remains asymptomatic during the first 20 years and may also remain so throughout life. However, when symptomatic, a significant number of these patients present with a significant loss in their quality of life and their clinical course is frequently worse due to the repeated episodes of cholangitis with the presence of intrahepatic calculi, intrahepatic abscesses, and sepsis [6]. The main consequences of congenital hepatic fibrosis are portal hypertension and the development of esophageal varices, which may result in hematemesis or melena [7]. In majority of patients, portal hypertension will not be present or will appear only later in the disease evolution. Bile stagnation and hepatolithiasis explain the recurrent cholangitis which dominates the clinical course and which is the principal cause of morbidity and mortality. Complications from Caroli syndrome are chronic abdominal pain, cholangitis, sepsis, choledocholithiasis, hepatic abscess, cholangiocarcinoma and portal hypertension (SS1) [7]. Cholangitis carries a poor prognosis with majority of patients dying within 5-10 years. The major published reports concerning bile ducts cysts have come from Great Britain, France, Japan and the United States. More than 200 cases of Caroli disease have been reported in literature and the incidence of Caroli syndrome is more than the pure form of Caroli disease [7]. In addition, various renal disorders may be seen in conjunction with these hepatic diseases, including autosomal polycystic kidney disease (both dominant and recessive forms), medullary sponge kidney and medullary cystic disease [8]. 

Laboratory findings are non-specific. Transaminases may be slightly elevated. The complete blood count may reveal thrombocytopenia and leucopenia if portal hypertension and hypersplenism are present. Elevated white blood cell count or ESR may indicate cholangitis. Blood urea nitrogen (BUN) and creatinine values should be obtained to detect associated renal disease. Demonstration of communication between sacculi and bile ducts is important in showing the Caroli disease component of Caroli syndrome, which can be achieved by US, CT scan, MRCP or endoscopic retrograde cholangiopancreatography (ERCP). Magnetic resonance imaging can suggest accompanying abnormalities such as portal hypertension, cirrhosis and renal involvement. Magnetic resonance is the most specific and non-invasive method to depict the multiple ductal dilatation seen in Caroli disease, called the “lollipop tree”, where cystic structures of different sizes, shapes and distribution freely communicate with the biliary tree [9]. ERCP is the gold standard in diagnosis in the case of fusiform dilatations of the biliary tracts. Congenital hepatic fibrosis is a histopathological diagnosis. The appearance of ductal dilatation can be confused with polycystic liver disease or obstructive bile duct dilatation [5]. It is well established that patients with Caroli syndrome suffer from relapsing episodes of cholangitis with the possibility of bacteremia and sepsis. It rarely presents in childhood and the diagnosis is usually made at an advanced age. Although, there are rare case reports published of even neonatal presentation of the disease [10-11].

In our patient, there were symptoms of the disease since childhood indicated by vomiting of blood and enlarged spleen but was not diagnosed towards the end of third decade of her life. The investigations revealed dilatation of intrahepatic bile ducts and huge splenomegaly with severe thrombocytopenia and severe leucopenia and ascites as prominent findings. This indicated the presence of portal hypertension most likely from congenital hepatic fibrosis. In this case, she presented with hematemesis in childhood and blood transfusion at the age 16 years and upper endoscopy proven presence of esophageal varices. All these suggested the likelihood of Caroli syndrome. After shunt surgery and splenectomy, there was remarkable hematological improvement. Caroli disease confers an approximately 7% risk of malignancy [12]. The treatment depends on the clinical features and the location of the biliary abnormality. When disease is localized to one hepatic lobe, hepatectomy relieves the symptoms and appears to remove the risk of malignancy. In diffuse Caroli disease, liver transplantation remains the treatment of choice. The usual complications of Caroli disease/syndrome are cholestasis, cholangitis, choledocholithiasis and cholangiocarcinoma, which were not found in this patient.

## Conclusions

Though infrequently encountered, Caroli syndrome should be considered an important differential diagnosis in the cases of chronic cholestasis where the exact etiology is undetermined. Most of the cases reported in the literature are from more affluent countries rather than developing countries like Tanzania. A higher degree of vigilance is required amongst physicians, especially in the countries with limited resources, so that sparsely available and expensive advanced diagnostic modalities can be used more effectively to yield a timely and accurate diagnosis.
